# Work engagement, emotional exhaustion, and OCB-civic virtue among nurses: a multilevel analysis of emotional supervisor support

**DOI:** 10.3389/fpsyg.2023.1249615

**Published:** 2023-10-26

**Authors:** Sabine Pohl, Abdel Djediat, Jan Van der Linden, Caroline Closon, Maura Galletta

**Affiliations:** ^1^Department Work and Consumption Psychology, Faculty of Psychology, Université Libre de Bruxelles, Brussels, Belgium; ^2^Department of Medical Sciences and Public Health, Faculty of Medicine and Surgery, University of Cagliari, Cagliari, Sardinia, Italy

**Keywords:** work engagement, emotional exhaustion, OCB-O, supervisor emotional support, multilevel analysis

## Abstract

**Introduction:**

This study investigates the moderating role of supervisor emotional support at the group level on the relationship between emotional exhaustion and work engagement with organizational citizenship behavior-civic virtue (OCB-civic virtue) at the individual level among nurses.

**Method:**

A cross-sectional study was carried out on 558 nurses nested in 36 working units from two hospitals in Algiers. A multilevel analysis using Hierarchical Linear Modeling was performed.

**Results:**

Results show that the positive effect of work engagement on OCB-civic virtue was moderated by supervisor emotional support at group level. The nurses emotional exhaustion and OCB-civic virtue negative relationship at the individual level is buffered by supervisor emotional support at group level.

**Discussion:**

In consequence, supervisor emotional support experienced by the team has an influence on the emotional exhaustion and work engagement OCB-civic virtue relationship.

## Introduction

Organizational citizenship behavior toward the organization (OCB-O) is an important resource for organizations and has a great impact on the quality of work that is carried out (Klotz et al., 2018). In the context of healthcare, nurses provide continuous care to hospitalized patients, assisting them to maintain or recuperate their health (van Doorn et al., 2016). Nurses fulfill their roles by delivering high-quality care services. Organizational citizenship behaviors are primordial aspects of nurses' quality of care. Indeed, prescribed job descriptions are not sufficient to include all that is expected at the workplace (Keyko et al., 2016). Literature highlights that experiencing emotional exhaustion negatively affects OCB-O (García-Sierra et al., 2016; Khan et al., 2019). Conversely, work engagement is found to be encouraging OCB-O (Bakker et al., 2012; Ginsburg et al., 2016; Slåtten et al., 2022). Although researchers have provided evidence that emotional exhaustion and work engagement influence OCB-O, to the best of our knowledge, no study has examined the role of supervisor emotional support as a resource in buffering these relationships. To address this gap, we propose to investigate the moderating role of supervisor emotional support at the group level in the relationships between emotional exhaustion, work engagement, and civic virtue, a subdimension of organizational citizenship behavior (OCB-civic virtue) which is an important dimension of OCB-O (Williams and Anderson, 1991). The present study contributes to the existing literature in the following ways. First, we provide a deep understanding of organizational citizenship behavior toward the organization (OCB-O) by focusing on how work engagement and emotional exhaustion are linked to OCB-civic virtue. Second, our findings emphasize the role of supervisor emotional support at the group level in buffering these relationships.

## Theoretical background

In work organizations, an important dimension of performance includes contextual performance such as OCB. It is defined as “performance that supports the social and psychological environment in which task performance takes place” (Organ, 1997, p. 95). OCB embraces discretional behaviors that are performed by employees to increase organizational effectiveness. OCB includes behaviors that go beyond the prescribed formal tasks (Urbini et al., 2020). OCB involves behaviors such as being altruistic at work or engaging in added tasks and keeping up with organization activities (Organ, 1997; Malingumu et al., 2016). Williams and Anderson (1991) suggest making a distinction between OCB that is focused on helping particular individuals (OCB-I) and OCB that is focused on the organization such as civic virtue (OCB-civic virtue). Of the different components of OCB (altruism, conscientiousness, courtesy, sportsmanship, and civic virtue), civic virtue is the one that is the most clearly focused toward the organization (Robinson and Morrison, 1995; Altuntaş et al., 2021). Indeed, civic virtue can be defined as “behavior on the part of an individual that indicates that he/she responsibly participates in, is involved in, or is concerned about the life of the company” (Podsakoff et al., 1990, p. 115). OCB-civic virtue belongs to the behaviors that foster the organization's reputation (Paillé and Boiral, 2013). In healthcare contexts, OCB-civic virtue concerns behaviors that promote the hospitals' image. OCB-civic virtue also reports to impact nurses's motivation to improve the way the team operates. OCB-civic virtue is associated empirically to workplace proactivity, task performance, and quality of care (Ginsburg et al., 2016). Because they are aimed at increasing organizational efficiency, OCB-civic virtue may be particularly critical in resource-restricted settings such as healthcare settings (Berta et al., 2018).

### Emotional exhaustion and OCB-civic virtue

Emotional exhaustion describes “feelings of being emotionally overextended and exhausted by one's work” (Wright and Cropanzano, 1998, p. 486). Emotional exhaustion is also frequently identified as the central stress dimension of burnout (Yulita and Abdullah, 2022). Employees who suffer from emotional exhaustion struggle to meet professional requirements. According to exchange theory (Blau, 1964), employees develop social exchange relationships to the extent that they receive benefits and rewards from their jobs and organizations. Jobs that generate emotional exhaustion are likely to disrupt these conditions. Emotional exhaustion, because it is often seen as unfair, hampers the development of qualitative social exchange relationships with the organization. Exhausted workers can perceive organization's actions that drive them to the point of emotional exhaustion as unfair (Cropanzano et al., 2003). They also identify less deeply with their organization which may drive unwillingness to engage in discretionary behavior that could benefit their organization. Employees who report high levels of emotional exhaustion develop a low level of organizational identification which may stimulate their averseness to engage in organizational citizenship behaviors, which is a behavior that benefits the organization (De Clercq et al., 2018). Social exchange may not be the only mechanism for the relationship between emotional exhaustion and OCB-civic virtue. According to the COR theory (Hobfoll, 2001), when a loss of resources appears, workers may conserve what is remaining to preserve themselves from future resource loss. Exhausted workers may remove non-obligatory activities such as OCB-O to conserve resources in favor of more central job activities (Liu et al., 2022).

Prior studies indicate a negative relationship between emotional exhaustion and OCB-O (Cropanzano et al., 2003; De Clercq et al., 2018). Emotional exhaustion was negatively associated with OCB-O but not with OCB-I. Employees assign emotional exhaustion produced by work principally to the organization. In healthcare settings, emotional exhaustion is considered as an organization-induced malady. Thus, to reestablish balance and preserve their resources, nurses suppress organizational citizenship behavior from which the organization benefits. By diminishing OCB-O, nurses can redeploy their resources on their job obligations (Tourigny et al., 2012).

Accordingly, our first hypothesis is that emotional exhaustion is negatively related to OCB-civic virtue.

*Hypothesis 1*. Emotional exhaustion is negatively associated with OCB-civic virtue.

### Work engagement and OCB-civic virtue

Work engagement concerns the amount of energy and enthusiasm employees feel toward their job (Bakker et al., 2012). When employees report high levels of engagement, they devote themselves into their work role and tend to embrace a wider perception of that role. Consequently, they are disposed to work above and beyond what is required of them (Christian et al., 2011). Furthermore, engaged employees feel indebted to their organization which keeps them contented. It is this feeling that drives the engaged employees to help their organization to expand (Gupta et al., 2017). When employees are happy about their work experience, they often give back via behaviors that are not necessarily prescribed but promote the organization's efficacy (Wan et al., 2018). Engaged employees are more willing to perform better and act positively in their organization and are thereby willing to perform OCB-O (Urbini et al., 2020). In addition, engaged employees are more willing to demonstrate OCB-civic virtue because of their engagement in a positive cycle of input and rewarding outcomes (Pohl et al., 2019). Engaged employees are more likely to exhibit organizational citizenship behavior toward the organization rather than toward their co-workers as their motivation for engagement stems from their job responsibilities. Interestingly, very few research studies have explored the existence of a positive relationship between OCB-I and work engagement (Gupta et al., 2017). Empirical evidence suggests a positive relationship between work engagement and OCB-civic virtue (Schaufeli et al., 2006; Alfes et al., 2013; Ginsburg et al., 2016; Park, 2019). Therefore, we developed the following hypothesis:

*Hypothesis 2*. Work engagement is positively associated with OCB-civic virtue.

### Supervisor emotional support at the group level as a moderator

It is essential for employees to communicate and control their own emotions during their exchanges at work (Karimi et al., 2013). Supervisors can give employees emotional support to help them regulate their emotions. Emotional support includes consideration, caring, and boost behaviors (Pohl and Galletta, 2017). Emotional supervisor support is an important work situational resource (Wang, 2022).

Most of the research studies have centered on the individual perception of emotional supervisor support, but employees from the same team may share their perceptions about the degree to which the supervisor takes them into consideration as there may be a collective perception of supervisor support (Vera et al., 2016). The collaboration between team members generates the shared perception of emotional supervisor support. Supervisor support at the group level contributes to a supportive group climate (Li et al., 2017). Emotional supervisor support at the group level provides employees with additional resources to better achieve their goals and enables cooperation among team members (Pohl and Galletta, 2017). In line with the conservation of resources theory (Hobfoll, 2001; Halbesleben et al., 2014), supervisor emotional support at the group level can be considered as an important resource that can minimize resource losses induced by emotional exhaustion. Following this perspective, we hypothesize the following:

*Hypothesis 3*. Supervisor emotional support at the group level moderates the relationship between emotional exhaustion and OCB-civic virtue at the individual level. The negative relationship between emotional exhaustion and OCB-civic virtue is weaker under high supervisor emotional support as opposed to low supervisor emotional support.

According to social exchange theory (Blau, 1964), employees give back to their organization by exhibiting more OCB-civic virtue when they perceive high support from their supervisor. A social support framework suggests that when supervisor emotional support is low, the impact of work engagement on employees' OCB-civic virtue can be mitigated. However, when supervisor emotional support is high, work engagement would have a stronger positive effect on employees' OCB-civic virtue. Thus, supervisor emotional support can be considered as a source of social support that moderates the effect of work engagement on OCB-civic virtue.

**Figure 1:**
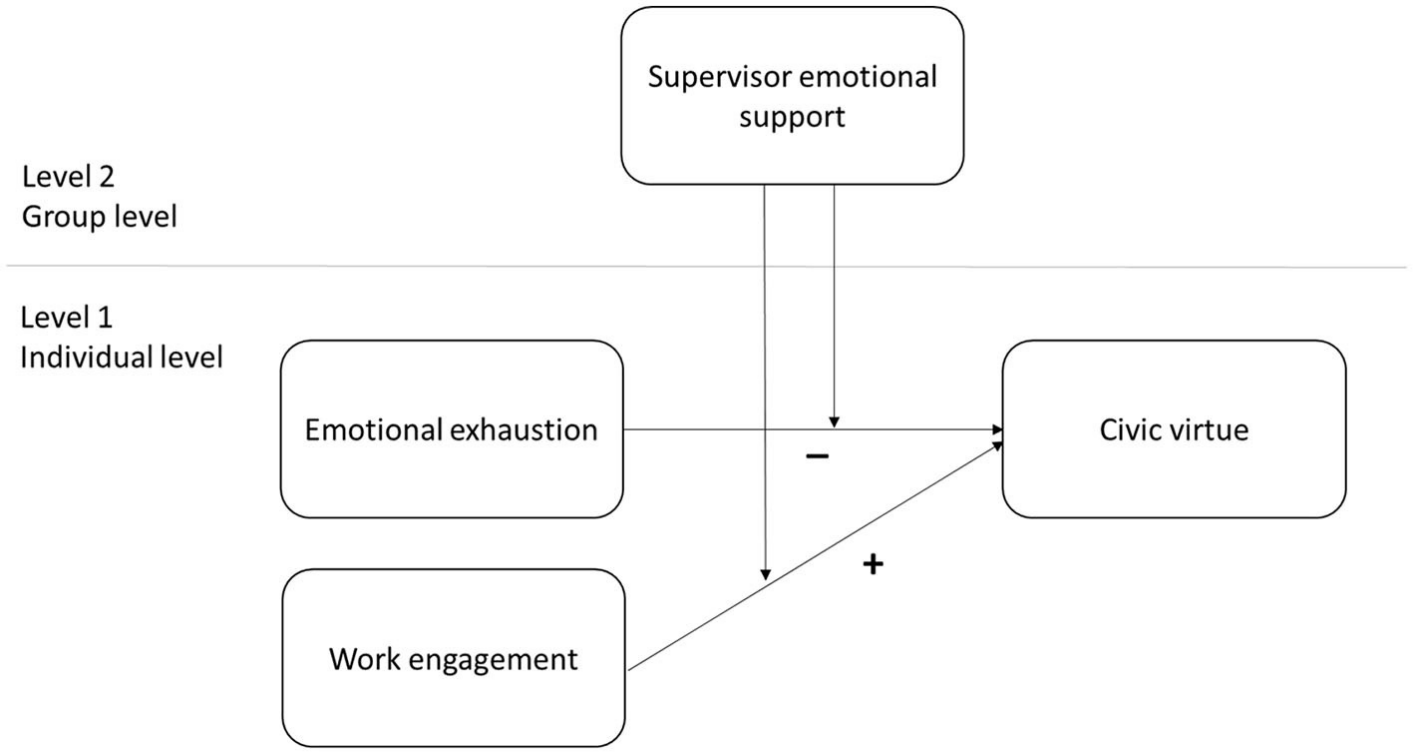
Cross-level moderation model framework.

*Hypothesis 4*. Supervisor emotional support at the group level moderates the relationship between work engagement and OCB-civic virtue at the individual level. The positive relationship between work engagement and OCB-civic virtue is stronger under high supervisor emotional support as opposed to low supervisor emotional support. Figure 1 resumes this cross-level moderation model.

## Materials and methods

### Study design

This study was led in the two largest public university hospitals in Algiers (Algeria). This research included a cross-sectional study proposal with self-reported questionnaires. Nurses were invited, on a voluntary basis, to complete a paper questionnaire available in two languages: French and Arabic. We followed a translation-back-translation process (Brislin, 1980) to translate the items into Arabic and into French. The distribution of the survey was carried out at the workplace with the assistance of the nurses' coordinators. The respondents completed the survey at home and then dropped it into one of the boxes situated at the hospitals. Confidentiality and anonymity were guaranteed for all participants.

### Measures

#### Emotional exhaustion

Emotional exhaustion was measured with the Maslach Burnout Inventory nine-item emotional exhaustion scale, validated in French by Truchot et al. (2012). This scale is designed to assess the degree of emotional exhaustion in a work situation and proposes items such as “I feel frustrated by my work.” The answers were given using a 5-point Likert scale ranging from 1 which means very rarely/never to 5 which is very often.

#### Work engagement

We used the UWES-9 item scale (Schaufeli et al., 2006). Each item was rated on a five-point scale. An example of an item is “At my job, I feel strong and vigorous.” The Likert scale score ranged from 1 (strongly disagree) to 5 (strongly agree).

#### Supervisor emotional support

We used the four-item supervisor emotional support scale developed by Pohl and Galletta (2017). Each item was rated on a five-point scale. For example, one item stated “My superior is genuinely concerned about my emotional wellbeing.” The Likert scale score ranged from 1 (strongly disagree) to 5 (strongly agree).

#### OCB-civic virtue

OCB-civic virtue was captured using the three-item civic virtue scale developed by Podsakoff et al. (1990). A sample item was “I attend professional events where my presence is encouraged but not formally required.” The Likert scale score ranged from 1 (strongly disagree) to 5 (strongly agree).

### Data analysis

#### Measurement model

To test our measurement model, a confirmatory factor analysis (CFA) was performed with Mplus 8. The CFA was carried out via structural equations modeling (SEM) using the Tucker–Lewis index (TLI), comparative fit index (CFI), and root mean square error of approximation (RMSEA) to test the quality of measurement model. Based on Kline (2005)'s suggestions, a good model fit is obtained when TLI and CFI are higher than or equal to 0.90, and RMSEA is ≤0.08. Factor validity was examined by comparing a hypothesized four-factor model (that includes work engagement, emotional exhaustion, OCB-civic virtue, and supervisor emotional support as four different factors) with a one-factor model in which all the items of the studied variables loaded on a common factor. The reliability of the scales was tested through McDonald's omega. The cutoff value for acceptability is ≥0.70.

To test the cross-level model, the hierarchical linear modeling (HL M) approach was used (Raundenbush and Bryk, 2002). Although emotional supervisor support was considered as a variable at the group level, it was measured through a self-reported questionnaire. Therefore, to statistically sustain emotional supervisor support as a shared variable at the group level (working unit), the scores of the variable were aggregated to the group level by creating a cluster variable. Following Klein et al. (2000), the aggregation was performed by examining within-group agreement and between-group variability. Intraclass correlation coefficients (ICC) were used to compute between-group variability. ICC(1) represents the reliability of individual ratings within each group. ICC(2) refers to the reliability of the mean group. The acceptable value for ICC(1) is close to or above 0.20 (Bliese and Castro, 2000). The recommended cutoff value for ICC(2) is close to or above 0.60 (Schneider et al., 1998). Lastly, *r*_wg_(*j*) with critical values was calculated for measuring the within-group agreement. The ideal value for *r*_wg_(*j*) is close or above 0.70 (Chen et al., 2004).

#### Testing the hypotheses

As a first step, we performed the null baseline model without the individual- or group-level predictors to analyze whether between-group variance in the predicted variable was significant or not. This model provided information about the amount of variance connected to individuals within and between different working units (Galletta et al., 2016). The random effect (τ_00_) was computed only for the intercept.

#### Cross-level interaction

The cross-level interaction was performed by following the method of Hofmann et al. (2000). We investigated whether supervisor emotional support at the group level (SES GL) moderated the effects of both emotional exhaustion (EE) and work engagement (WE) on civic virtue (CV) at the individual level. In particular, two models were tested for exploring the cross-level interaction. A first preliminary model analyzed the relationship between both WE and EE and CV at the individual level. With this model, the variability of the effects of the two predictors (WE and EE) across teams was measured. A second model analyzed the cross-level moderating effect of SES GL on the relationship between both WE and EE and CV (Hypotheses 3 and 4). By splitting the total variance of both WE and EE into their within- and between-group components, we investigated which source of variance interacted with SES GL. To test the nature of the interaction, we followed Aiken and West's (1991) method. Regression lines were plotted for the relationships between both WE and EE and CV at low and high levels of SES GL.

### Ethics approval

The approval to carry out the research was obtained by the Ethical Committee of the lead university according to the Code of Ethics of the World Medical Association. The certificate number was 007/2016. Written informed consent was obtained from participants before to complete the questionnaire.

## Results

### Participants

Nurses who had over 6 months of experience were included in the study. Participants in the study were 558 nurses, representing a participation rate of 38%. Participants were nested in 36 working units (surgery, pediatrics, oncology, etc.) from two hospitals in Algiers. Each nurse works in a unit, supervised by a nursing supervisor. The mean age was 37.6 years (SD ± 8.11 years), with an average job experience of 14.45 years (SD ± 13.53 years), and 78.9% were women.

### Descriptive statistics

Means, standard deviations, and correlations among the variables are displayed in Table 1. The findings show acceptable reliability for all the research variables.

**Table 1:** Means, standard deviations, correlations, and reliabilities.

	***M***	**SD**	**1**	**2**	**3**	**4**
1. WE	3.65	1.17	(0.97)			
2. EE	2.82	1.17	−0.585^**^	(0.95)		
3. SES GL	3.18	1.15	0.631^**^	−0.568^**^	(0.98)	
4. CV	3.26	1.34	0.368^**^	−0.322^**^	0.290^**^	(0.94)

WE, work engagement; EE, emotional exhaustion; SES GL, supervisor emotional support at group level; CV, civic virtue.

McDonald's omega values are presented in parenthesis. ^**^p < 0.01.

### Measurement model

The overall goodness-of-fit for the model with four factors was adequate: chi-square (χ^2^) = 1,283.86; df = 312; *p* < 0.001; comparative fit index (CFI) = 0.94; Tucker–Lewis index (TLI) = 0.93; root mean square error of approximation (RMSEA) = 0.08. The comparison of the four-factor model with the one-factor model showed a significant worsening of the fit: χ^2^ (df = 324,) = 8,430.38, CFI = 0.51, TLI = 0.47, and RMSEA = 0.24.

### Intraclass correlation

Scale scores for SES suggested high agreement and significant reliability. The ICC(1) coefficient was 0.49, and the ICC(2) value was 0.91. The average *r*_wg_(*j*) for SES across 36 work units was 0.69 (median = 0.87), with a critical value (group size mean = 10) of 0.61. These results showed that considering SES as a group-level variable is acceptable.

### Testing the hypotheses

The results from the null model showed that within-group variation for CV was significant [random effect = 0.34, χ^2^ (35) = 71.34, *p* < 0.001 and ICC(1) = 0.11], thus showing that 11% of variance in CV resides between groups. This result indicates a nesting effect in the data and legitimizes cross-level analyses.

Hypothesis 1 postulated that, at the individual level, WE was positively associated with CV (γ_10_ = 0.33, *p* < 0.001). Hypothesis 2 supposed that, at the individual level, EE was negatively associated with CV (γ_20_ = −0.16, *p* < 0.01). The results provided support to this hypothesis.

#### Cross-level interaction

**Figure 2:**
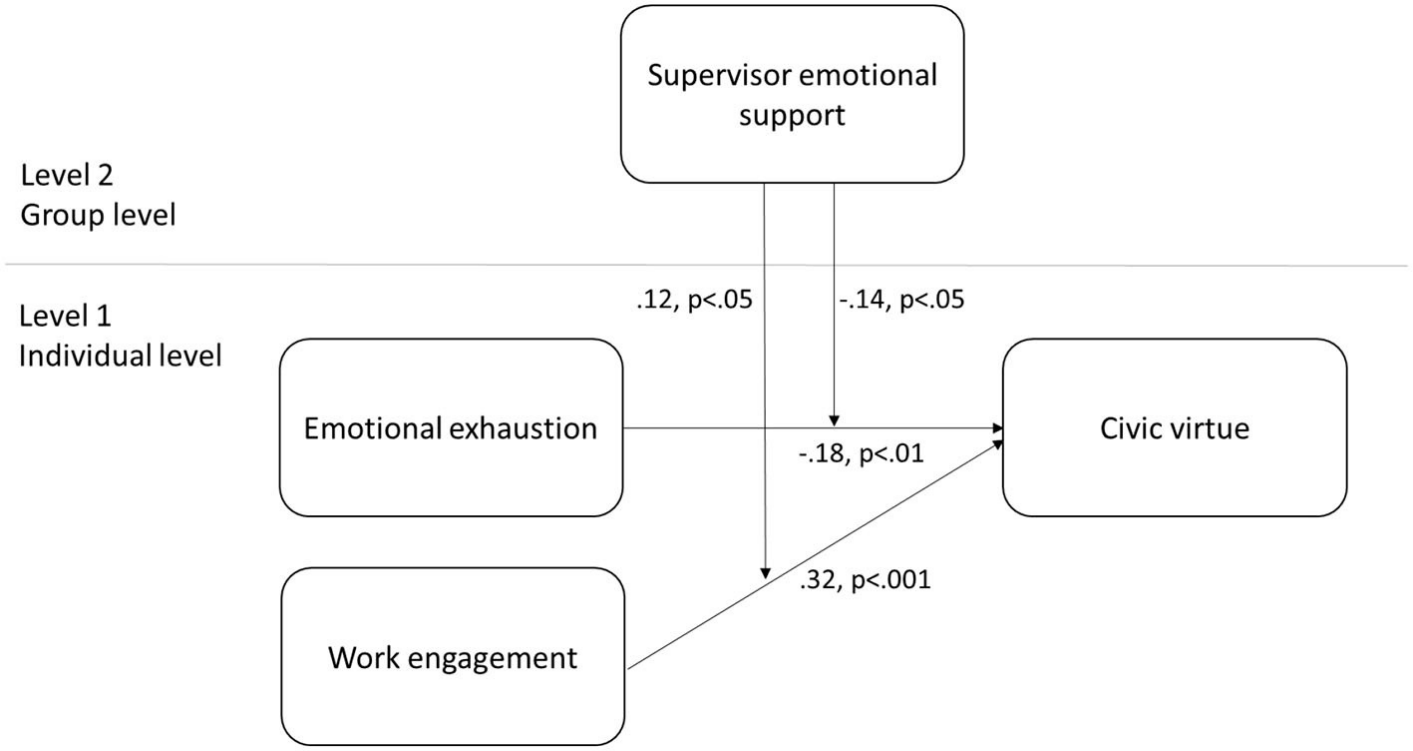
Cross-level moderation model.

We examined whether the interaction effects between both WE and EE at the individual level and SES GL were significant. SES GL was inserted as a predictor variable of the variance in the slopes relating both WE and EE and CV at the individual level. The results revealed a non-significant interaction between groups (γ_01_ = 0.13, *T*-ratio = 1.37, *p* > 0.05) and a significant cross-level interaction effect between SES GL and WE (γ_11_ = 0.12, *T*-ratio = 2.23, *p* < 0.05) and between SES GL and EE (γ_21_ = −0.14, *T*-ratio = −2.12, *p* < 0.05), thereby supporting both Hypotheses 3 and 4 (Table 2 and Figure 2).

**Table 2:** Cross-level interaction results.

**Dependent variable: civic virtue**	**Preliminary model**		**Moderation effect**	
	**Coefficient**	**SE**	**Coefficient**	**SE**
**Level 1—Individual-level**				
Intercept (γ_00_)	3.28^***^	0.09	3.28^***^	0.08
Work engagement (γ_10_)	0.33^***^	0.06	0.33^***^	0.06
Emotional exhaustion (γ_20_)	−0.16^**^	0.07	−0.18^**^	0.06
**Level 2—Cross-level interaction**				
Work engagement^*^Supervisor emotional support (γ_11_)			0.12^*^	0.05
Emotional exhaustion^*^Supervisor emotional support (γ_21_)			−0.14^*^	0.07
**Variance components**				
Variance in L1 (δ^2^)	1.33		1.33	
Variance in L2 (τ_00_)	0.18		0.15	
Variance in L2 slope (τ_11_)	0.02		0.01	
Variance in L2 slope (τ_21_)	0.04		0.03	

N = 558; work units = 36.

^***^p < 0.001.

^**^p < 0.01.

^*^p < 0.05.

In Level 1 analysis, both work engagement and emotional exhaustion were group-mean centered.

**Figure 3:**
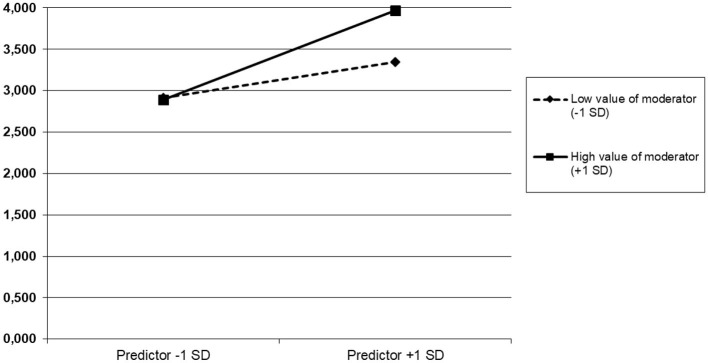
Cross-level moderation effect of emotional supervisor support on work engagement–civic virtue relationship. Predictor: work engagement; moderator: emotional supervisor support; dependent variable: civic virtue.

**Figure 4:**
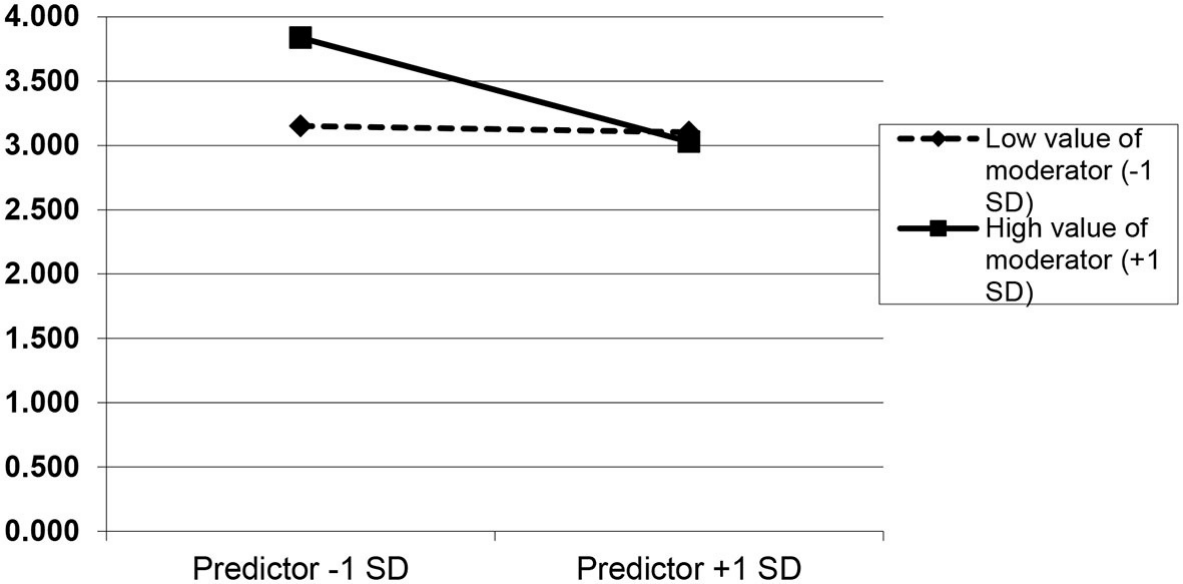
Cross-level moderation effect of emotional supervisor support on emotional exhaustion–civic virtue relationship. Predictor: emotional exhaustion; moderator: emotional supervisor support; dependent variable: civic virtue.

The interaction was as expected. However, we found that the positive relationship between WE and CV was significantly more robust when SES GL was high (slope for high values of moderator = 0.46, *t* = 8.42, *p* < 0.001; slope for low values of moderator = 0.18, *t* = 3.38, *p* < 0.01). In other words, nurses who perceived high levels of work engagement and emotional support from their supervisors were more likely to engage in behaviors of civic virtue toward their working unit (Figure 3). The negative relationship between EE and CV was significantly the strongest when SES GL was high and EE was low (slope for high values of moderator = −0.34, *t* = −6.30, *p* < 0.001; slope for low values of moderator = 0.02, *t* = −0.38, *p* > 0.05). This means that nurses who perceived high levels of emotional support from their supervisors and low levels of emotional exhaustion had high civic virtue behaviors, and *vice versa*, with low levels of supervisor emotional support and high or low levels of emotional exhaustion, and civic virtue was unchanged (Figure 4).

## Discussion

This research study contributes to the existing literature by analyzing the relation between emotional exhaustion, work engagement, and nurses's OCB-civic virtue. In the present research, we hypothesized that emotional exhaustion was negatively associated with OCB-civic virtue and that work engagement was positively associated with OCB-civic virtue. Moreover, we hypothesized that supervisor emotional support at the group level buffered these relationships. Consistent with our predictions and previous studies (Cropanzano et al., 2003; Tourigny et al., 2012; De Clercq et al., 2018), our results revealed that emotional exhaustion is negatively related to OCB-civic virtue. Emotional exhaustion diminished OCB-civic virtue through both social exchange and resource-based processes. When job demands exceed personal resources, exhausted workers may assign their emotional exhaustion to the organization. Furthermore, in line with COR theory, the energy-depleting effect of emotional exhaustion drives employees to preserve resources to prevent additional resource losses. Workers might be too exhausted to commit in OCB-civic virtue (Liu et al., 2022).

Consistent with the results of previous studies (Schaufeli et al., 2006; Alfes et al., 2013; Gupta et al., 2017; Urbini et al., 2020), our findings indicate that work engagement is positively related to OCB-civic virtue. The more the nurses are engaged in their work, the more the nurses display OCB-civic virtue. Nurses who are energetic and enthusiastic are more likely to exhibit OCB, specifically in the form of civic virtue. These results corroborate the COR theory (Hobfoll, 2001), and resource gains from engaged employees have been translated into OCB-civic virtue.

Moreover, supervisor emotional support at the group level is found to be a significant moderator between work engagement, emotional exhaustion, and OCB-civic virtue, which is another important contribution of this study. The findings highlight that the negative relationship between emotional exhaustion and OCB-civic virtue and the positive relationship between work engagement and OCB-civic virtue are buffered by emotional supervisor support at the group level. More precisely, high levels of work engagement that a nurse invests in his/her work are associated with high levels of OCB-civic virtue when emotional supervisor support at the team level is high. Engaged nurses develop more OCB-civic virtue if they feel that they are emotionally supported by their supervisor. Furthermore, high levels of emotional exhaustion are less associated with low levels of OCB-civic virtue when emotional supervisor support at the team level is high. Our results extend existing research by showing that the relationship between both work engagement and emotional exhaustion and OCB-civic virtue can be modified by team characteristics. Supervisors take part in supportive behaviors that are not only directed toward specific individual workers but also toward their team. Perceptions of supervisor emotional support shared among nurses who work in the same work unit, work engagement, and emotional exhaustion impact OCB-civic virtue. Therefore, the research study contributes to literature by testing a hierarchical linear model in which both individual-level and group-level variables affect employees' OCB-civic virtue. These cross-level interaction effects suggest that a worker's working experience can be influenced by work unit-specific characteristics such as the quality of supervisor emotional support.

## Limitations

Although the study provides new knowledge about the topic, some limitations must be acknowledged. First, the use of a cross-sectional design prevents us from making causal statements regarding the relationship between variables. Longitudinal studies should be performed to investigate the long-term effects of work engagement, emotional exhaustion, and supervisor emotional support on OCB-civic virtue to increase the generalizability of the study. Future research should examine the relationship between supervisor emotional support on other important working performance factors such as proactive work behavior (innovation, change, and creativity). Another limitation includes the use of a self-report to collect the data, which may have intensified the relationships between variables. Future study designs should integrate other methods of data collection—especially those including OCB—such as the assessment from supervisors.

However, a strength of this study is that it is one of the first analyzing the relationship between work engagement, emotional exhaustion, supervisor emotional support, and OCB-civic virtue at different organizational levels. In this sense, the study contributes making additional value to the knowledge in nursing management.

## Conclusion and practical implications

The main findings of the study show that the negative relationship between emotional exhaustion and OCB-civic virtue and the positive relationship between work engagement and OCB-civic virtue are buffered by emotional supervisor support at the group level. Emotional supervisor support help workers to cope with emotional exhaustion. However, it is important that employers may take action to prevent emotional exhaustion before it appears. From a practical point of view, the multilevel perspective allows for interventions at both the individual and group levels (Bliese and Jex, 2002). At the individual level, organizations should foster work engagement among employees and reduce emotional exhaustion to promote civic virtue behavior. Effective interventions to reduce emotional exhaustion may include avoiding excessive workload by implementing flexible work schedules, clarifying task division, and replacing absent colleagues. To promote work engagement, some interventions based on setting and pursuing personal goals are useful.

Managers should consider supervisor emotional support at the group level as a resource for enhancing civic virtue behaviors and should foster supervisors to acquire this emotional supportive behavior. Interventions to increase supervisor emotional support may include trainings for supervisors about how to provide emotional support and how to enhance communication skills for fostering collaboration between supervisors and employees. Furthermore, supervisors should stimulate teamwork and build a climate that encourages supportive behavior and emotional support.

## Data availability statement

The original contributions presented in the study are included in the article/supplementary material, further inquiries can be directed to the corresponding author.

## Ethics statement

The approval to carry out the research was obtained by Ethical Committees of the lead university according to the Code of Ethics of the World Medical Association. The certificate number was 007/2016. Written informed consent was obtained from participants before completing the questionnaire.

## Author contributions

Study design: SP and AD. Data collection: AD. Data analysis, study supervision, and manuscript writing: SP and MG. Proof reading: JV and CC. All authors contributed to the article and approved the submitted version.
